# Left atrial appendage occlusion in ventricular assist device patients to decrease thromboembolic events: A computer simulation study

**DOI:** 10.3389/fphys.2022.1010862

**Published:** 2022-09-29

**Authors:** Mojgan Ghodrati-Misek, Thomas Schlöglhofer, Christoph Gross, Alexander Maurer, Daniel Zimpfer, Dietrich Beitzke, Francesco Zonta, Francesco Moscato, Heinrich Schima, Philipp Aigner

**Affiliations:** ^1^ Center for Medical Physics and Biomedical Engineering, Medical University of Vienna, Vienna, Austria; ^2^ Ludwig Boltzmann Institute for Cardiovascular Research, Vienna, Austria; ^3^ Department of Cardiac Surgery, Medical University of Vienna, Vienna, Austria; ^4^ Department of Biomedical Imaging and Image Guided Therapy, Medical University of Vienna, Vienna, Austria; ^5^ Institute of Fluid Dynamics and Heat Transfer, Technical University of Vienna, Vienna, Austria

**Keywords:** atrial fibrillation, sinus rhythm, left atrial appendage occlusion, thromboembolic risk, ventricular assist device, computational fluid dynamics

## Abstract

Atrial fibrillation (AF) is a common comorbidity in left ventricular assist device (LVAD) patients and has been identified as a risk factor for thromboembolic stroke. Blood stagnation within the left atrial appendage (LAA) is considered a possible major source of thrombosis and clinical studies have shown reduced thromboembolic risk after LAA occlusion (LAAO). Therefore, this study aims to investigate the effect of LAAO on thrombosis-related parameters using patient-specific simulations. Left ventricular and left atrial geometries of an LVAD patient were obtained from computed tomography and combined with hemodynamic data with either sinus rhythm (SR) or AF generated by a lumped parameter model. In four simulations applying contractile walls, stagnation volume and blood residence times were evaluated with or without AF and with or without LAAO. Reduced atrial contraction in AF resulted in unfavorable flow dynamics within the left atrium. The average atrial velocity was lower for the AF simulation when compared to SR, resulting in a 55% increase in the atrial stagnation volume (from 4.2 to 6.5 cm^3^). Moreover, blood remained in the LAA for more than 8 cardiac cycles. After LAAO the atrial stagnation decreased from 4.2 to 1.4 cm^3^ for SR and from 6.5 to 2.3 cm^3^ for the AF simulation. A significant stagnation volume was found in the LAA for both SR and AF, with larger values occurring with AF. These regions are known as potential sources for thrombus formation and can be diminished by LAAO. This significantly improved the thrombus-related flow parameters and may also lower the risk of thromboembolic events from the appendage.

## 1 Introduction

Left ventricular assist device (LVAD) therapy is considered a treatment for patients with end-stage heart failure and is used either as a bridge to transplant or as destination therapy (Mancini and Colombo, 2015). Despite the success of this treatment (Molina et al., 2021), a high risk for thrombosis and, consequently, stroke related mortality persists (Acharya et al., 2017; DeVore and Stewart, 2017).

The left atrial appendage (LAA) has been considered as a potential source of thrombus formation for atrial fibrillation (AF) patients because of their reduced atrial contractility, which can lead to blood stasis and platelet deposition (Blackshear and Odell, 1996). AF has also been shown to be a significant risk factor for thromboembolic events (Stulak et al., 2013; Deshmukh et al., 2017) affecting up to 54% of patients undergoing LVAD implantation (Deshmukh et al., 2017).

Occlusion of the LAA has been associated with a lower risk of thromboembolic events in AF patients undergoing cardiac surgery (Friedman et al., 2018). For the LVAD population, LAAO has been recommended to reduce the prevalence of thromboembolic events, even for patients free from AF (Deshmukh et al., 2019), however the rationale remains hypothetical.

Computational Fluid Dynamics (CFD) has the potential to provide some insight into the changes occurring within the heart chambers after LAAO. Individual CFD simulations can be performed with patient-specific models of LVAD patient’s hearts, which can be used to investigate the influence of both AF and LAAO on haemodynamics and blood flow. While in several computational studies, the intraventricular flow pattern during LVAD support was modeled without including the essential motion of the heart wall (Chivukula et al., 2018, 2019; Liao et al., 2018a; Liao et al., 2018b; Ghodrati et al., 2020; Ghodrati and Khienwad, 2021; Ghodrati and Schlöglhofer, 2021), To date, no CFD simulation study has examined the effects of AF on the contractile left heart or of LAAO in patients with LVAD support.

In this study, a patient-specific flow-field simulation was performed in which the contraction of the left atrium and left ventricle was implemented by moving the endocardial wall of these two chambers. This was used to 1) evaluate the blood flow patterns for an LVAD patient with sinus rhythm (SR) and with AF and 2) to investigate the post-procedure blood flow dynamics to quantify the efficacy of LAA occlusion on thrombus-related flow parameters.

## 2 Materials and methods

### 2.1 Patient models

**FIGURE 1:**
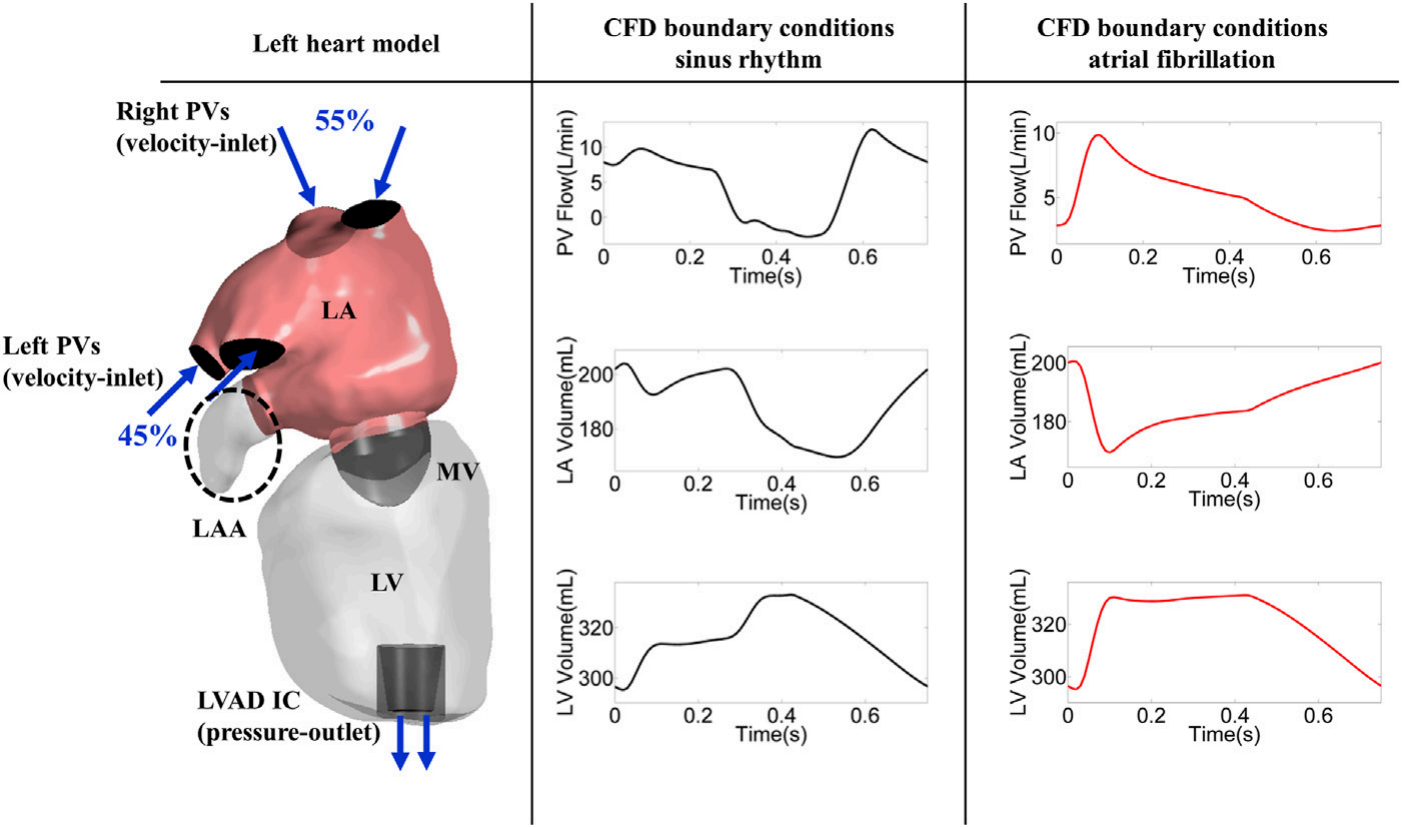
Left heart model (encircled dashed ellipse marks LAAO) and CFD boundary conditions; PV: pulmonary vein, LA: left atrium, LV: left ventricle, MV: mitral valve, LAA: left atrial appendage, SR: sinus rhythm, AF: atrial fibrillation, LVAD: left ventricular assist device, IC: inflow cannula.

The left atrium and the left ventricle of an LVAD patient were segmented from computed tomography (CT) images using Mimics Research 20.0 and 3-matic Research 13.0 (Materialise, Belgium NV). The end systolic volume of the atrium and ventricle of this patient were 169 and 295 cm^3^, respectively (Figure 1). The left atrial appendage geometry was occluded virtually (Ansys, SpaceClaim 19.3, Pennsylvania, United States) (Figure 1).

A mitral valve model (Domenichini and Pedrizzetti, 2015) was defined using the following parametric equations:

$$x_{v}\left(\theta ,s\right)=R\cos\theta \left(1-s\cos\varphi \right)-\varepsilon Rs\cos\varphi$$

$$y_{v}\left(\theta ,s\right)=R\sin\theta \left(1-sk\cos\varphi \right)$$

$$z_{v}\left(\theta ,s\right)=-s^{2}\left(\frac{1+k}{2}+\varepsilon \cos\theta +\frac{1-k}{2}\cos2\theta \right)R\sin\varphi$$

In which θ is 100 points from 0 to 2π and s is 40 points from 0 to 1. ε=0.35 shows the symmetry ratio between anterior and posterior leaflet. k=0.6 , shows the ellipticity of the valvular edge. φ=60 , shows the opening angle of the mitral valve.

The created valve geometry was placed at the position and matching the orientation of the mitral valve as defined via the CT images.

The mitral valve was considered in the open status with a rigid wall and the flow rate over the mitral valve was controlled using the volume change of the left atrium and left ventricle, leading to zero flow rate over the mitral valve during systole.

### 2.2 Meshing

An unstructured tetrahedral mesh with a total of 1.5 million cells was created (Ansys Meshing, 19.3, Pennsylvania, United States). All of the mesh elements showed a skewness factor below 0.84 and orthogonal quality above 0.16, which is in the recommended ranges of the Ansys Meshing User Guide (Baker, 2016). A suitable mesh size was chosen based on a mesh independence study, which can be found in the Supplementary Material.

### 2.3 Haemodynamics

The haemodynamics values for a typical LVD patient under full support were determined based on previous studies, in which the mass flow rate over LVAD inflow cannula was 5.2 L/min, blood pressure was equal to 83.8 ± 10.5 mmHg and heartrate was 79.3 ± 17.0 bpm (Jacquet et al., 2011; Martina et al., 2013; Muthiah, 2015; Burrell et al., 2015; Haft et al., 2007). Moreover, the left ventricular inflow filling pattern, E/A ratio of 1.2 ± 0.7 was chosen from (Estep et al., 2014).

The haemodynamics were simulated using the previously developed and published lumped parameter model (Moscato et al., 2012). Briefly, the LPM of the circulatory system consists of active ventricles and atria, simulated by a non-linear elastance function, with heart valves to prevent backflow, compliant vessels and resistances for the pulmonary and systemic vascular bed (arterioles). The pump characteristics (pump head, pump speed and pump output) of the HVAD (Medtronic) were implemented with the inlet of the pump connected to the LV and the outlet of the pump in the aorta.

To simulate the hemodynamics for a typical LVAD patient, the following parameters in the original lumped parameter model were adjusted: 1) Heartrate was set to 80 bpm, LV peak iso-volumic pressure was reduced from 100 to 80 mmHg, 2) right ventricular peak iso-volumic pressure was reduced from 80 to 50 mmHg, 3) mean circulatory pressure was reduced from 12.5 to 10 mmHg, 4) arterial systemic resistance was reduced by 6%, and 5) To adapt the E/A ratio, the constant (alpha) for the calculation of the end-diastolic pressure-volume relation was increased by 70%.

For the atrial fibrillation scenario, the only change compared to the typical LVAD patients was the deactivated LA active contraction. The stroke volume for the AF simulation was a result of the passive contraction of the atrium (Figure 1).

### 2.4 Boundary conditions and solver setup

The Navier-Stokes equations were solved with a finite volume approach in the CFD solver (FLUENT, Ansys 19.3, Pennsylvania, United States) and blood flow was modeled using the Laminar method and Newtonian fluid with a density of 1,060 kg/m^3^ and a dynamic viscosity of 0.0035 Pa s was considered. The incoming flow was delivered over the right and left pulmonary veins with a distribution of 55 and 45%, respectively (Wong et al., 2014). The velocity and pressure boundary conditions were imposed at both the inlet and outlet.

The atrial and ventricular volume waveforms (Figure 1) were assigned through user-defined functions (UDFs) to implement the dynamics of the atrium and the ventricle. The atrial and ventricular volume waveforms were divided in 750 equal time-steps in one cardiac cycle. Combination of shape optimization algorithm and dynamic mesh was used to model the contraction of the LA and LV. The shape optimization is a feature in Ansys Fluent in which the geometry is changed in order to reach a specific criterion. The shape optimization algorithm was utilized to calculate the geometry deformation based on the volume difference between two consecutive time-steps. The calculated value then was used to move the mesh vortices using dynamic mesh in which a combination of smoothing and re-meshing methods. The dynamic mesh approach in ANSYS (Ansys Fluent, 19.3, Pennsylvania, United States) applies a form of smoothing in which the size of the existing elements is modified until the size/quality of the elements exceed the user defined thresholds (skewness below 0.84, size below 2 mm). In case of a large deformation, the solver relies on re-meshing, with which new mesh elements will be added to keep the size/quality in the defined range. The inlet and outlet boundaries as well as the mitral valve were considered rigid.

The PISO (pressure implicit with splitting of operators) algorithm was employed along with a second order upwind scheme. Simulations were performed for duration of 12 cardiac cycles with a temporal resolution of 0.001 s. The first four cardiac cycles were defined as the initialization phase to ensure fully developed atrial and ventricular flow after this time and were not considered in the final data evaluation. Convergence was observed in each time step when the residuals were below 10^−3^ for continuity, *x*-velocity, *y*-velocity and *z*-velocity. All simulations were performed on the Vienna Scientific Cluster using 2,500 core-hours for each simulation.

### 2.5 Flow parameter evaluation

The atrial and ventricular flow variation over time was determined using the standard deviation of velocity.

Wall shear stress (WSS) within the atrium and the ventricular surface was categorized in a low non-physiological range (0–0.2 Pa) (Rayz et al., 2010) and physiological range (0.2–9 Pa) (Ghodrati et al., 2020). Volumes with a time-averaged velocity of less than 1 mm/s were defined as stagnation volumes to highlight stasis regions (Dintenfass, 1964; Rayz et al., 2008).

Atrial and ventricular blood washout was quantified using a virtual ink technique (Rayz et al., 2010; Prisco et al., 2017) in which all fluid domains were initialized with an ink concentration of 0, with a value of 1 at the inlets representing flow of fresh blood. The rate of atrial and ventricular washout was calculated by the percentage of old blood in the atrium and ventricle, normalized by the atrial and ventricular volume.

## 3 Results

### 3.1 Volume and flow curves

**FIGURE 2:**
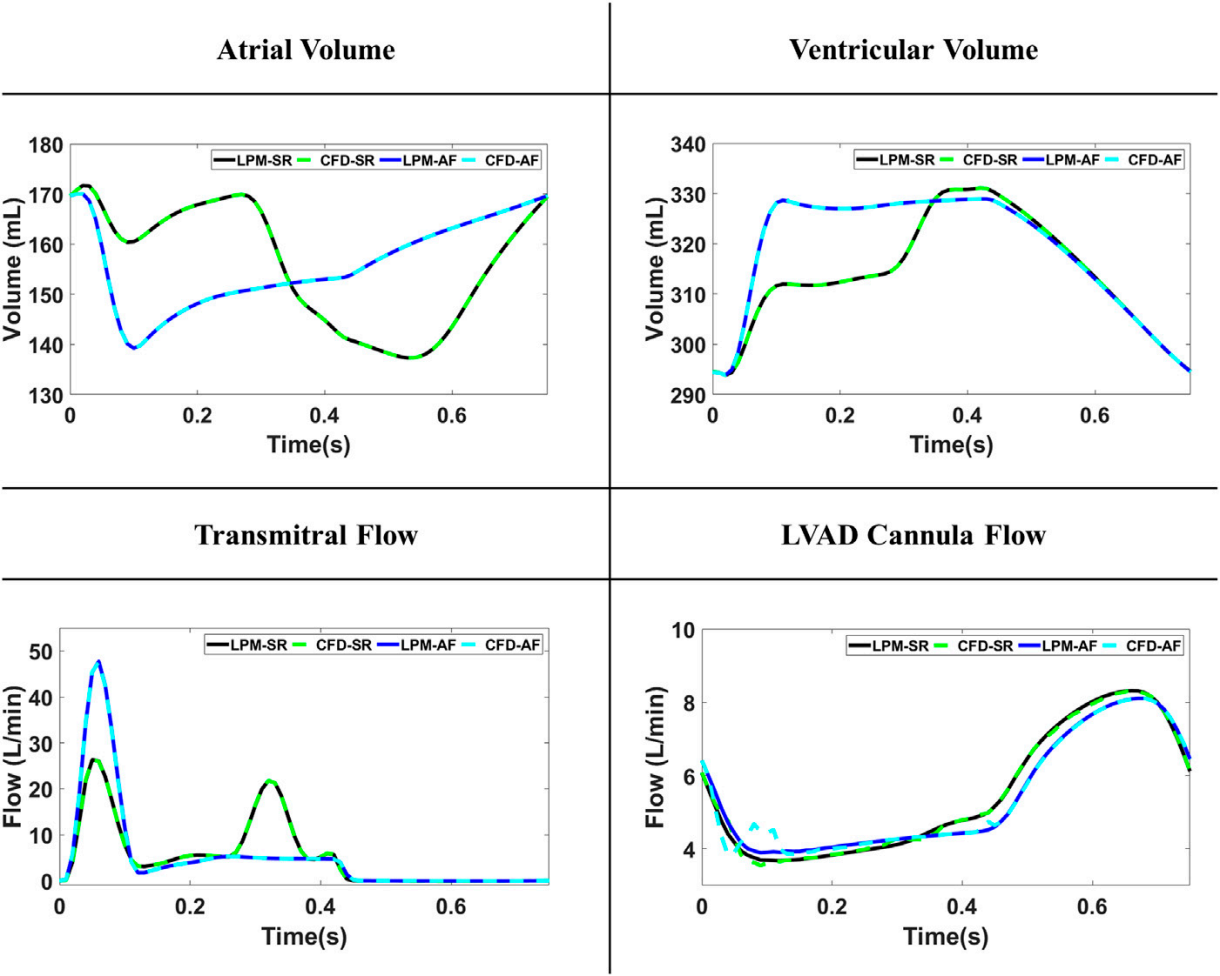
The volume and flow curves comparison between the lumped-parameter model (LPM) and computational fluid dynamics (CFD) models for simulations with sinus rhythm (SR) and atrial fibrillation (AF).

The volume changes of the LA and LV were accurately implemented by CFD simulation leading to identical flow over the mitral valve and LVAD cannula between CFD simulations and LPN model. A comparison of the volume changes and flow rate values between CFD and LPM results for the SR-LAA and AF-LAA simulation can be seen in Figure 2. Similar results were observed for two other simulations (SR-LAAO and AF-LAAO).

### 3.2 Blood flow patterns

Blood flow patterns with and without atrial fibrillation and with/without appendage occlusion were successfully simulated and on the first view showed similar results, however the detailed evaluation of the flow fields showed significant differences in thrombus-related flow parameters.

#### 3.2.1 SR versus AF

**FIGURE 3:**
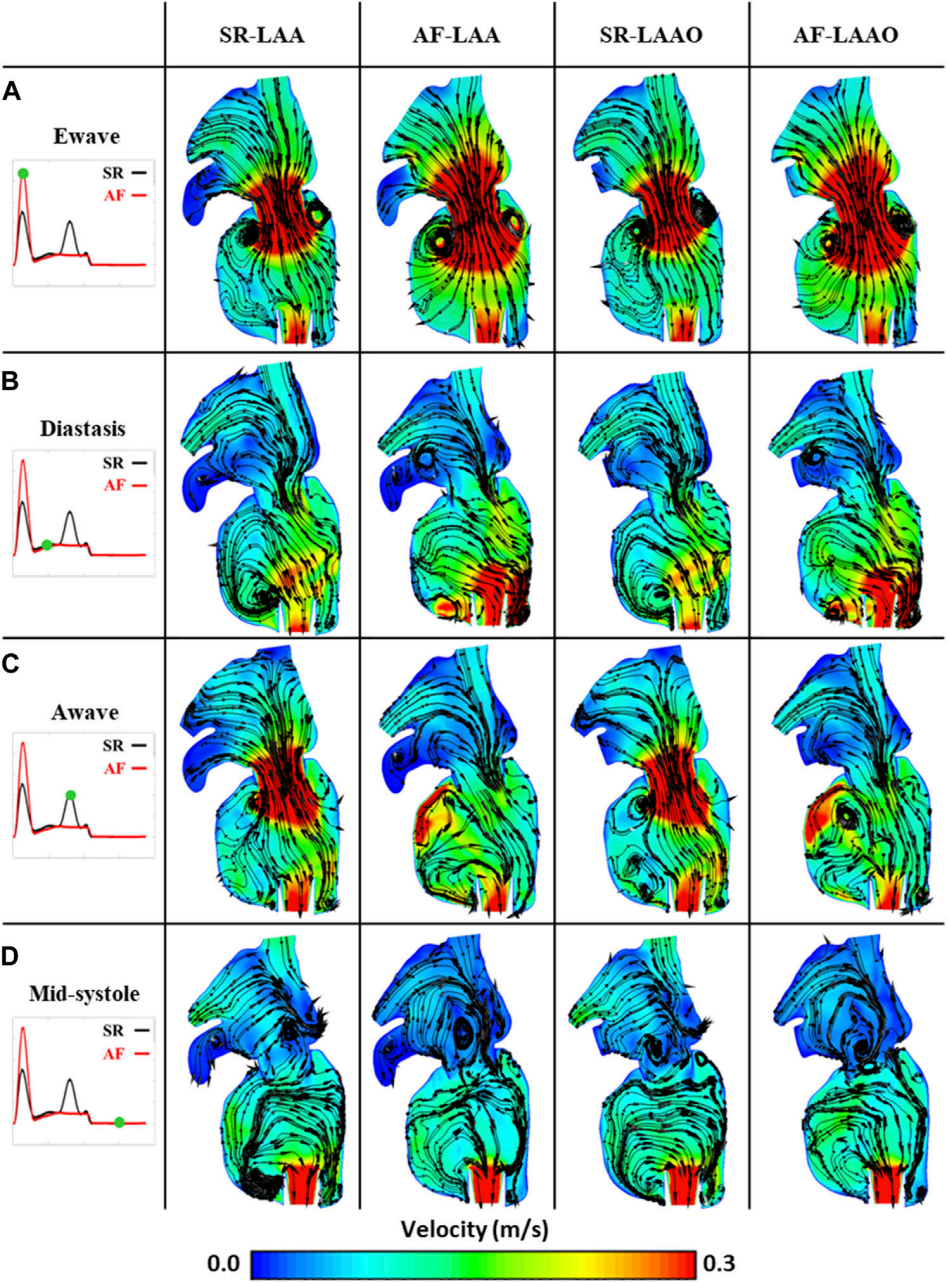
Phasic average flow fields at Ewave **(A)**, Diastasis **(B)**, Awave **(C)** and Mid-systole **(D)** in a coronal plane for SR-LAA, SR-LAAO, AF-LAA and AF-LAAO.

Atrial fibrillation led to a 10% (from 6.8 cm/s to 6.1 cm/s) reduction of the blood velocity within the atrium. At the E-wave, due to the passive contraction of the atrium, high blood velocity was created at the mitral valve, leading to rapid filling of the ventricle (Figure 3A). With the onset of the diastasis phase, formation of a recirculation zone was observed within the LAA for both SR and AF simulations (Figure 3B). During the A-wave this region diminished for the simulation with the sinus rhythm due to the left atrial kick (Figure 3C). In the AF simulation, persistence of the recirculation zone inside the LAA could be observed until the end of the cardiac cycle (Figures 3C,D).

#### 3.2.2 SR (LAA versus LAAO)

Occlusion of the appendage, which is a source of recirculation zones and stasis areas, improved the atrial haemodynamics. The average blood velocity within the atrium increased by 5%. The standard deviation of velocity in the LAA was five times lower than in the LA chamber (LAA: 1.1, LA: 6.2 cm/s).

#### 3.2.3 AF (LAA versus LAAO)

The results of the AF simulation pre and post LAAO were similar to the simulation with SR. A 6% increase in mean atrial velocity over one cardiac cycle was observed due to the LAAO. For the AF simulation, a six times lower standard deviation was observed within the LAA compared to LA (LAA: 1.1, LA: 7.3 cm/s).

### 3.3 Wall shear stress and stagnation volume

#### 3.3.1 SR versus AF

**FIGURE 4:**
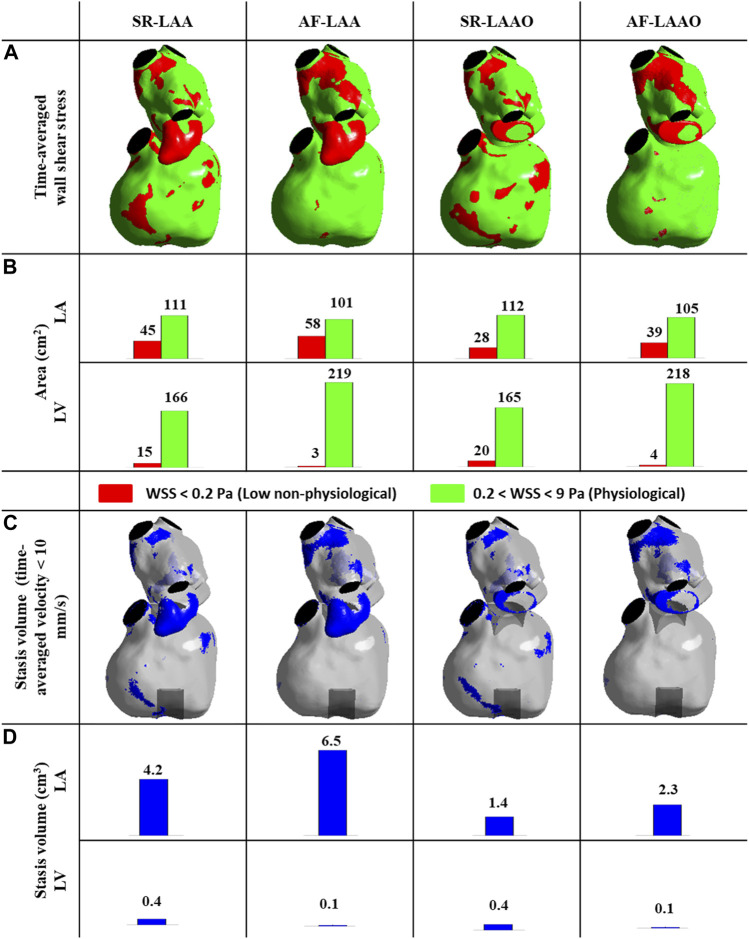
**(A,B)** Time-averaged wall shear stress distribution at the atrial and ventricular wall **(A,B)**, time-averaged stagnation volume within the atrium and ventricle **(C,D)**.

With either SR or AF with intact LAA, thrombosis-related parameters, mainly at the LAA, were found to be significant i.e., low WSS values and high stasis volumes. These parameters however had lower values for the simulation with SR. Atrial fibrillation leads to an increase of 29% (from 45 to 58 cm^2^) on the low WSS area (Figures 4A,B) and 55% (from 4.2 to 6.5 cm^3^) on the stasis volume (Figures 4C,D) compared to the SR simulation.

#### 3.3.2 SR (LAA versus LAAO)

Appendage occlusion resulted in a reduction of low WSS areas and stasis volumes; the reduction of 37% (from 45 to 28 cm^2^) in areas with low WSS (Figures 4A,B) and 66% reduction (from 4.2 to 1.4 cm^3^) in stasis volume was observed post-LAAO for SR (Figures 4C,D). The values within the ventricle remained similar pre- and post-LAAO for both SR and AF (Figure 4).

#### 3.3.3 AF (LAA versus LAAO)

Comparable results were also observed with AF; with a 32% (from 58 to 39 cm^2^) reduction in low WSS areas (Figures 3A,B) and 64% reduction (from 6.5 to 2.3 cm^3^) in stasis volume (Figures 4C,D). The LAAO did not influence the areas with low WSS or stagnation volume within the ventricle (Figure 4).

The effect of atrial fibrillation on thrombosis-related flow parameters (low WSS, stagnation volume) was mainly observed within the left atrium. These parameters were increased with atrial fibrillation, but were significantly decreased by occlusion of the atrial appendage leading to more favorable condition.

### 3.4 Blood washout

#### 3.4.1 SR versus AF

**FIGURE 5:**
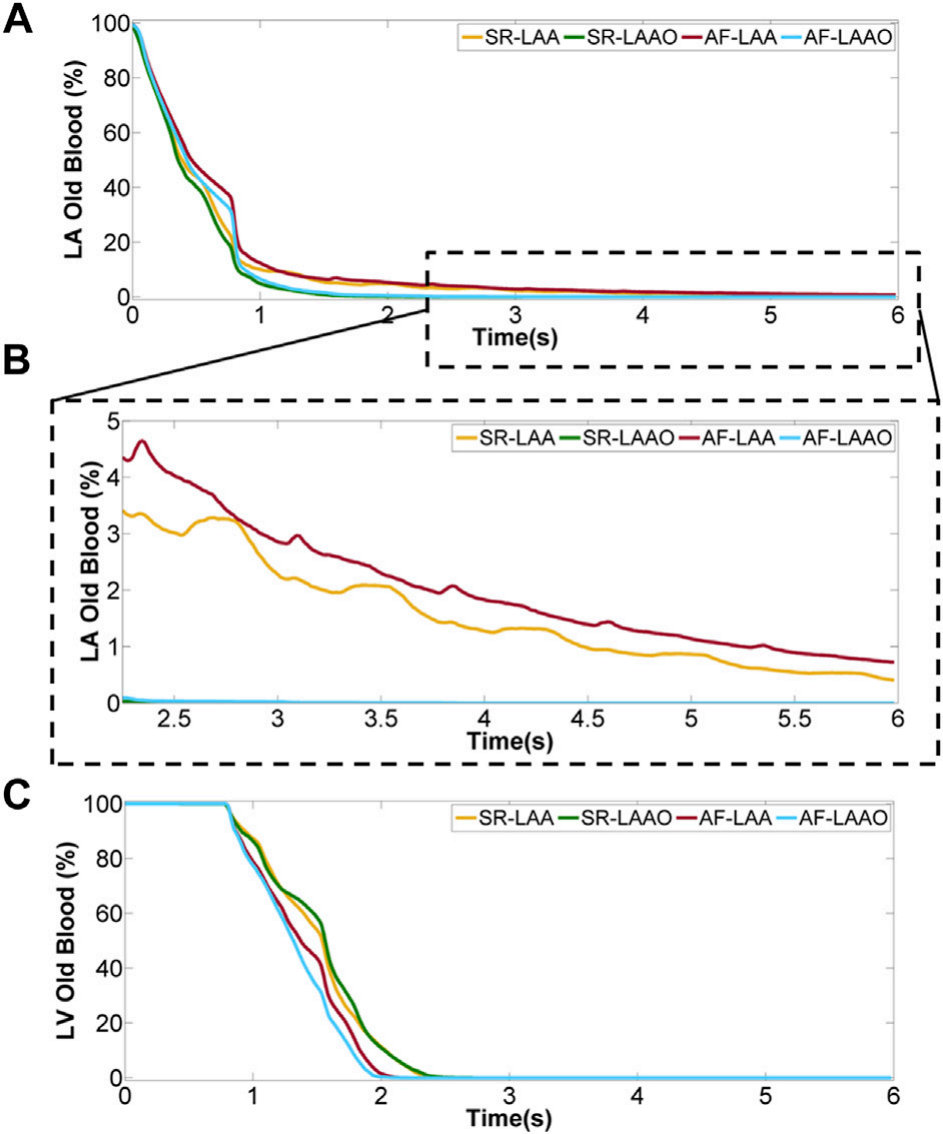
Percentage of old blood remaining within the atrium **(A)**, a close-up for last 5 cycles **(B)** and ventricle **(C)** over 8 cardiac cycles.

The blood washout which was calculated using virtual-ink method showed similar behaviour within the left atrium for SR and AF simulations before LAAO, after 3 cardiac cycles around 95% of the old blood was replaced with new blood. However, the ventricular washout was slightly different. The complete replacement of the old blood with fresh blood in the LV took 2.5 s in the SR simulation and 2.2 s in the AF simulation (Figures 5A,B).

#### 3.4.2 SR (LAA versus LAAO)

Occlusion of the appendage significantly accelerated the replacement of the old blood with new blood within the atrium (Figures 5A,B), while the blood volume exchange within the ventricle remained similar for pre- and post-LAAO (Figure 5C). After 2.5 s the old blood within the atrium was replaced with new blood for LAAO while without the occlusion 3% (5 ml) of the old blood entering the LA on the first cycle remained in the LAA.

The evaluation of the blood washout only within the appendage showed that after 8 cardiac cycles, 8% of the old blood entering the LAA in the first cardiac cycle remains there.

#### 3.4.3 AF (LAA versus LAAO)

**FIGURE 6:**
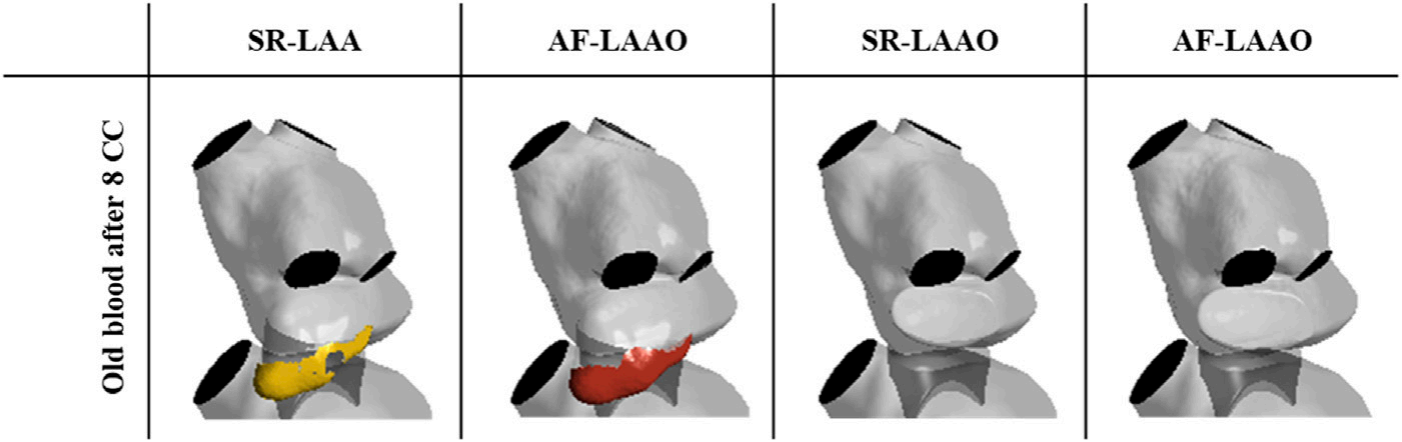
Volume of the old blood remaining in the LAA after 8 cardiac cycles.

A similar behaviour as with sinus rhythm was observed for the atrial fibrillation after LAAO (Figure 5). After 3.2 s the replacement of the old blood with fresh blood within the atrium was observed for the LAAO simulation while without the occlusion 4% (6 ml) of the old blood remained in the LAA. Evaluation of the washout within the appendage showed that from the 9 ml of blood entering the LAA on the first cycle, 14% of 9 ml resides there after 8 cardiac cycles (Figure 6).

## 4 Discussion

Clinical studies show high prevalence of AF for LVAD patients. AF was diagnosed in 26–54% of patients before LVAD implantation (Stulak et al., 2013; Deshmukh et al., 2017) and 13–28% post-LVAD implantation (Hickey et al., 2016; Deshmukh et al., 2018). Preoperative AF is associated with an increased risk for thromboembolic events after LVAD implantation (Stulak et al., 2013; Enriquez et al., 2014), while postoperative AF increases the risk of ischemic stroke, and device thrombosis in the long term after LVAD implantation (Deshmukh et al., 2018). Surgical LAAO at the time of LVAD implantation was linked to a decrease in thromboembolic events which has been shown to be true with and without AF (Deshmukh et al., 2019), however, no clinical trial data is currently available on this issue to support the decision making process with respect to left atrial appendage procedures (Deshmukh et al., 2017).

This is the first study in which numerical simulations show the effect of LAAO on flow patterns in the left heart under LVAD support. Four simulations were performed in total: two with normal sinus rhythm (with and without LAA) and two with AF (with and without LAA). The hemodynamic differences and various thrombosis-related parameters including stasis volume, low WSS and blood residence time were computed (Rayz et al., 2010). Since fluid dynamical parameters and its influence on the mechanism for thrombus formation are still poorly understood, various parameters with complementary roles were considered to evaluate the risk of thrombosis. Low wall shear stresses show the areas on the wall where the blood in their vicinity experiences very low velocities and could be the origin of the thrombus formation. However, defining a sharp threshold bears the risk to oversimplify mechanisms and consequently on detecting the size of stasis regions. Therefore, the stagnation volume was used for complementary evaluation of the high-risk regions which shows the high-risk areas within the cavity. Also, the residence time was used to demonstrate the locations where blood resides there for a long time, which in combination with low blood velocity, might lead to platelet aggregation.

Reduction in the flow velocities of the left atrium and LAA were observed for AF when compared to SR. This behavior was also observed in 4D flow MRI analysis and was associated with an elevated risk of stroke for AF patients (Markl et al., 2016). A flow recirculation zone was observed in the LAA with low velocity, leading to the formation of a stagnation volume for both SR and AF simulations. The WSS also remained abnormally low along the LAA wall during the entire simulation and part of the initially injected fluid remained within the LAA for more than 8 cardiac cycles. An increased flow residence time in a region with low shear stresses and low velocity values may promote blood clot formation and subsequent adhesion to the LAA wall, since the formed clot is not transported away by the flow. The results of this study highlight the risk of clot formation in the LAA for LVAD patients independent of AF. This could be the reason for the lower prevalence of thromboembolic events for patients who received surgical LAAO at the time of LVAD implantation (Deshmukh et al., 2019). This finding is consistent with findings of clinical studies performed for patients with AF who were scheduled to undergo cardiac surgery. In this study LAAO showed larger influence than antithrombotic therapy among high-risk AF patients with regards to risk reduction of ischemic strokes and systemic embolism (Chen et al., 2021; Whitlock et al., 2021).

The distribution of low WSS at the ventricular wall and thus the formation of stagnant volume within the ventricular cavity was unchanged for both SR and AF, for both occluded and unoccluded LAAs. These results show that thrombosis-related parameters are important in the LAA, but play a minor role in the left ventricle.

### 4.1 Limitation

The evaluation was performed for one patient geometry that allows us comparison between the sinus rhythm and atrial fibrillation with and without LAA for LVAD patients in the same geometry. However, There are other forms and shapes of the LAA (Cactus, Chicken Wing, Windsock, and Cauliflower) which likely influence the hemodynamics within the LA (Beigel et al., 2014). Since the quality of CT scans for LVAD patients was not sufficient for segmentation of the mitral valve and papillary muscles, a standardized parametric mitral valve was used which allows comparison with other studies (Liao et al., 2018a; Liao et al., 2018b).

## 5 Conclusion

To conclude, this is the first study that applies moving walls and realistic hemodynamics in order to quantitatively analyze the effects of AF and LAAO in the LVAD population. The findings of this study highlight the unfavorable hemodynamics at the LAA for LVAD patients with both SR and AF. Occlusion of the LAA significantly reduced the thrombus related flow mechanical parameters. This procedure could be performed concomitant with LVAD implantation and might lower the risk of thromboembolic events caused by the appendage.

## Data Availability

The original contributions presented in the study are included in the article/Supplementary Material, further inquiries can be directed to the corresponding author.

## Abbreviations

AF, Atrial fibrillation; CFD, Computational Fluid Dynamics; CT, Computed tomography; LA, Left atrium; LAA, Left atrial appendage; LAAO, Left atrial appendage occlusion; LV, Left ventricle; LVAD, Left ventricular assist device; SR, Sinus rhythm; WSS, Wall shear stress.
