# Supplementary material for: Left atrial appendage occlusion in ventricular assist device patients to decrease thromboembolic events: A computer simulation study
Source: Front Physiol. 2022 Sep 29;13:1010862. doi: 10.3389/fphys.2022.1010862 (PMC9557157; doi:10.3389/fphys.2022.1010862)
Supplement: Supplementary file 1 [file DataSheet1.PDF]

Supplementary Material

Mesh independence study

The mesh independency study was performed for three different mesh sizes for the simulation of the left atrium with the sinus rhythm refers as SR-LAA in the manuscript. To this end, the mesh size was changed from 2 mm to 0.8 mm for the surface and from 4 to 1.6 mm for the volume. This sizing results to Mesh 1 with 500 k, Mesh 2 with 1.1 million and Mesh 3 with 2.1 million elements (Supplementary Figure 1).

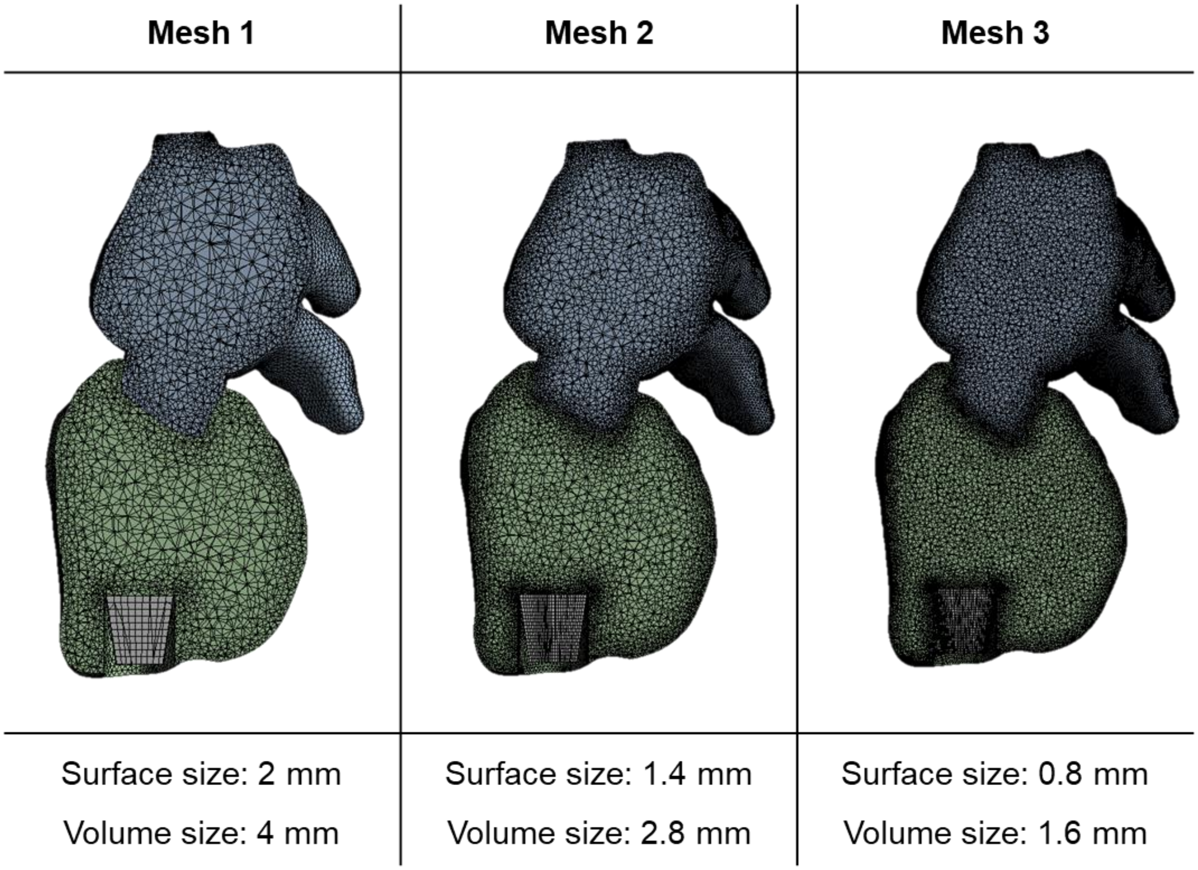

Supplementary Figure 1. CFD meshes created for mesh independency study showing at a coronal cross-section of the left heart (top row) and the size of the mesh elements (bottom row).

In order to evaluate the transient behavior of the simulations the Weighted Mean Relative Difference (WMRD) was calculated over one cardiac cycle based on the (Equation), (Sonntag et al., 2018):

$$WMRD\% = 100 \times \frac{\sum_t |p_t - p'_t|}{\sum_t (p_t + p'_t)/2} \qquad 1 \qquad \text{Equation 1}$$

Where p is the time dependent parameter in mesh size 1 and p' is the time dependent parameter in mesh size 2.

The WMRD was calculated for the average and max velocity magnitude within the Left Atrium (LA) and Left Ventricle (LV) as well as the max and average Wall Shear Stress (WSS) on the LA wall and LV wall. The results of different mesh comparison can be seen in Supplementary Table 1.

Supplementary Table 1. Mesh independence study parameters

Parameter of interest	Mesh 1 & mesh 2	Mesh 2 & mesh 3
**LA average velocity magnitude WMRD [%]**	10	2
**LA max velocity magnitude WMRD [%]**	8	1
**LV average velocity magnitude WMRD [%]**	3	2
**LV max velocity magnitude WMRD [%]**	3	3
**LA wall average WSS WMRD [%]**	14	2
**LA wall max WSS WMRD [%]**	17	12
**LV wall average WSS WMRD [%]**	5	3
**LV wall max WSS WMRD [%]**	26	7

Small differences were seen between Mesh 2 (1.1 million) and Mesh 3 (2.1 million), therefore the Mesh 2 was considered for this study.

1. Sonntag SJ, Zebrowski E, Neidlin M, Hugenroth K, Benkowski R, Motomura T, et al. Virtual Fitting and Hemodynamic Simulation of the EVAHEART 2 Left Ventricular Assist Device and Double-Cuff Tipless Inflow Cannula. ASAIO J [Internet]. 2018 Sep 19 [cited 2018 Sep 25];Online First. Available from: https://journals.lww.com/asaiojournal/Abstract/onlinefirst/Virtual_Fitting_and_Hemodynamic_Simulation_of_the.98818.aspx
